# Interaction between *Salmonella* and Schistosomiasis: A Review

**DOI:** 10.1371/journal.ppat.1005928

**Published:** 2016-12-01

**Authors:** Amber Hsiao, Trevor Toy, Hye Jin Seo, Florian Marks

**Affiliations:** 1 International Vaccine Institute, Department of Epidemiology, Republic of Korea; 2 Technische Universität Berlin, Department of Health Care Management, Berlin, Germany; University of Pennsylvania, UNITED STATES

## Abstract

The interaction between schistosomiasis and *Salmonella* is a particularly important issue in Africa, where dual infection by the parasite and the bacterium are likely common. In this review, the ways in which schistosomiasis affects human biology as it relates to *Salmonella* are described. Those who are infected by both organisms experience reduced immunological functioning, exhibit irreversible organ damage due to prolonged schistosomiasis infection, and become latent carriers of *Salmonella enterica* serotypes Typhi and Paratyphi and *S*. Typhimurium. The sequestration of the bacteria in the parasite leads to ineffective antibiotic treatment because the bacteria cannot be completely killed, and lingering infection may then lead to antimicrobial resistance. These manifestations are likely not just for those dually infected but also for those first infected with schistosomes and, later, *Salmonella*. More data are needed to better understand dual infection, particularly as it may impact treatment and prevention of schistosomiasis and *Salmonella* in sub-Saharan Africa.

## Introduction

Substantial progress has been made in reducing the incidence of communicable diseases globally. In Africa, where the burden of communicable diseases is particularly high, the mortality rate of children under five years of age has dropped from 173 to 95 per 1,000 live births from 1990 to 2012 [1]. This reduction in deaths among children under five are a result of a combination of efforts, including increased access to clean water, sanitation, and improved hygiene, as well as increased rates of immunizations for vaccine-preventable diseases.

There are, however, still a number of preventable diseases that are neglected, including *Salmonella* and schistosomiasis. While each of these diseases has a significant impact on morbidity and mortality in sub-Saharan Africa, coinfection has important implications for country control and prevention plans. The following review summarizes the current state of knowledge on the biological interactions between *Sal*. *enterica* subtypes Typhi and Paratyphi and invasive non-typhoidal *S*. Typhimurium (iNTS) and schistosomiasis, as well as the burden of each in the context of sub-Saharan Africa.

### Burden of *Salmonella* in Africa

Typhoid and paratyphoid fever are major causes of morbidity and mortality globally, causing an estimated 21.7 million illnesses and 217,000 deaths annually [2, 3]. Invasive nontyphoidal salmonellosis also contributes significantly to the global burden of disease, with an estimated 3.4 million cases and a case fatality rate of 20% [4]. Yet, data specific to the African region are limited. *Salmonella* serotypes Typhi (*S*. Typhi) and Paratyphi A, B, and C, are the best described serotypes that cause typhoid fever (TF) and paratyphoid fever, respectively [5]. Both serotypes of the bacteria act by invading the small intestine and entering the bloodstream and other organs (e.g., the liver and spleen) where they multiply further before re-entering the bloodstream. Clinical presentation varies from mild-grade fever to abdominal discomfort and complications including intestinal perforations [6]. Practically, the diagnostic gold standard generally involves performing a blood culture to isolate the bacteria. Once confirmed, treatment often consists of antibiotics [6]. Relapses can occur in 5%–10% of patients [7].

Despite a clear understanding of disease mechanisms and treatment, lack of diagnostic capabilities and surveillance systems, among other factors, has made it difficult to accurately describe the burden of TF (and paratyphoid fever) and iNTS disease in Africa. In 2008, the World Health Organization (WHO) expressed the need for more epidemiological data to estimate the incidence of *Salmonella* in Africa [3]. Few studies have characterized the burden in this region. Mogasale et al. estimates incidence from 123 per 100,000 in West Africa, to 195 per 100,000 in South Africa, to as high as 465 per 100,000 persons in East Africa [8]. A systematic review of 25 studies found an incidence of 725 per 100,000 in sub-Saharan Africa [9]. Other country-specific estimates in Africa are similarly within these ranges [10, 11].

African countries will be better able to plan for *Salmonella* control as new data emerge, though other challenges will arise. Namely, there is increasing concern in the international community that infection of parasites, sometimes multiple parasites, may compromise the body’s ability to deploy a protective response to other acute bacterial or viral infections. Consequently, immunological responses to control and prevention strategies such as vaccinations may be attenuated in the presence of other infections commonly found in developing countries [12–14].

### Burden of Schistosomiasis in Africa

Africa also suffers from the highest burden of neglected tropical diseases (NTDs), including schistosomiasis (also known as bilharzia), an infectious chronic disease caused by parasitic worms found in fresh water [15]. There are five main species of the worm, but *Schistosoma mansoni* and *S*. *haematobium* are predominantly found in Africa and manifest in the form of intestinal and urogenital schistosomiasis, respectively [16]. Schistosomiasis ranks second only to malaria amongst cases of parasite-associated mortality, killing an estimated 280,000 people annually in Africa alone [17]. In 2013, more than 40 million people were treated for schistosomiasis, though at least 261 million people required preventive treatment, >90% of whom live in Africa [15]. The few schistosomiasis seroprevalence studies that exist indicate that 90% of those living in high-risk areas will acquire an infection at some point in their lives, which often occurs before the age of ten. In low-risk areas, between 25% to 40% will be infected by the age of 35 [18, 19]. Fragile health systems coupled with lack of safe water, sanitation, and hygiene in many areas throughout Africa make schistosomiasis particularly challenging to control [20].

Clinical underdetection of schistosomiasis is common. The current gold standard diagnostic is microscopic examination of stool or urine for eggs [21], though it is not sensitive enough to detect mild infections where adult worms have not produced eggs in numbers sufficient for detection. Some studies have found that upwards of 20%–30% of active infections are missed in stool or urine samples, as eggs are not always passed into the specimen on the day of testing [22–24]. Thus, the reported prevalence of schistosomiasis is likely to be an underestimate. By some estimates, the burden of active cases of schistosomiasis in 2007 may have been between 391 to 587 million people worldwide [12].

Schistosomiasis can also cause long-term effects beyond the period of active infection. For example, infections have been found to be significantly associated with anemia, chronic pain, diarrhea, exercise intolerance, and malnutrition [25]. The parasite causes acute granulomatous and fibrotic injury to bodily organs that can lead to chronic disability. Based on a meta-analysis of 135 randomized and observational studies of schistosomiasis, the parasitic infection is associated with a number of disability-linked morbidities. Repeated cycles of infection are common in endemic areas where infection prevention and control measures (including control of intermediate hosts) are lacking. If left untreated, the worms live within the host for an average of two to five years. Frequently, however, those at risk of multiple waves of infection will generally experience active infection for 15 or more years on average [26]. Schistosomiasis can be treated with praziquantel, which kills the adult worms in the body [27]. Treatment with praziquantel is generally safe, even in those living with decreased immune functioning, such as those with HIV [28]. Thus, in high transmission areas, WHO recommends that treatment be repeated yearly for a number of years, and cases monitored to determine treatment effectiveness. The greatest limitation to schistosomiasis control, however, is availability of praziquantel [16]. While efforts have been made to improve access, including the use of cheaper generics, WHO estimates that only 13.1% of people globally who required praziquantel treatment received it [15]. Lai et al. estimate that 123 million doses of praziquantel are needed per year to treat school-aged children, and 247 million are needed for all affected populations [29]. While access to treatment is important, control of schistosomiasis requires a multipronged strategy that involves interrupting the chain of transmission by ensuring that water is free of parasites and basic sanitation is adhered to, especially as drug resistance to praziquantel becomes more of an issue [30, 31].

### Interaction between *Salmonella* and Schistosomiasis

There has been limited information to date of the interaction between *Salmonella* and schistosomiasis. However, evidence suggests that this interaction warrants more attention, especially as additional evidence emerges about the true burden of typhoid fever and iNTS disease in Africa [32]. Based on available data, typhoid and schistosomiasis have similar geographic distributions, and the highest burden of both NTDs falls on children under 15 years of age [18, 19, 33]. While studies do exist that assess the immunological response to schistosomal and *Salmonella* coinfection, most have been conducted on small samples of patients in Egypt and Brazil in the late 1980s [34]. Few studies to our knowledge have been conducted in Africa (see Table 1) [35–39].

**Table 1:** Summary of studies on interaction between schistosomiasis and *Salmonella*.

Study	Year*	Location	Number of Subjects	Ages	Schistosomiasis†	*Salmonella* †	Main interaction described	
**Reduced immunological functioning**								
Higashi GI, et al.	1975	Egypt	13	12–30	*S*. *mansoni*, *S*. *haematobium*	*S*. Paratyphi A, *S*. Typhi	Renal functioning is particularly reduced in patients with *S*. *mansoni* and *S*. Paratyphi A infections
Carvalho EM,et al‡	1983	Brazil	38 (15 controls)	3–66	Schistosomiasis	*Salmonella*	Incidence of circulating immune complex was significantly higher in those with chronic salmonellosis than in patients with schistosomiasis alone
el-Hawy AM, et al.	1985	Egypt	55 (20 controls)	15–55	*S*. *mansoni*	*Salmonella*	*Salmonella* infection more difficult to treat in chronic salmonellosis with *S*. *mansoni*
Lambertucci JR, et al.	1988	Brazil	3	5 (*n* = 1) 14 (*n* = 2)	*S*. *mansoni*	*Salmonella*	Clinical evidence of renal lesions due to coinfection
Martinelli R, et al.	1992	Brazil	16 (divided into 2 groups by diagnosis)	17.7 (mean age of Group I) 23.0 (mean age of Group II)	Schistosomiasis	*Salmonella*	Prolonged *Salmonella* infection causes renal damage that exacerbates pre-existing schistosomal glomerulopathy
Abdul-Fattah MM, et al.	1995	Egypt	190 (50 controls)	No ages reported	Schistosomiasis	*Salmonella*	Coinfection leads to greater risk of developing glomerulonephritis, primarily due to prolonged *Salmonella* bacteremia
**Schistosomiasis patients as latent carriers of *Salmonella***								
Halawani A, et al.	1960	Egypt	36 (divided into 3 groups by diagnosis and treatment)	17–45	*S*. *haematobium*	*Salmonella*	Chloramphenicol less effective in presence of urinary schistosomiasis; treating schistosomiasis first found to increase response to chloramphenicol
Hathout Se-D	1970	Egypt	54	10–50	*S*. *haematobium*	*Salmonella*	Incidence of typhoid urinary carrier state much higher in children and young adults than older ages
Young SW, et al.	1973	Egypt	2	No ages reported	*S*. *mansoni*	*S*. Paratyphi A	*S*. Paratyphi cultured from *S*. *mansoni* in patients with chronic salmonellosis
Franco MM, et al.	1976	Zimbabwe	142 (divided into 4 groups by diagnosis)	No ages reported	*S*. *mansoni*, *S*. *haematobium*	*S*. Typhi	Patients with bilharziasis had higher Widal positivity than those without for *Salmonella*
Ali OF, et al.	1978	Egypt	200	10–40	*S*. *haematobium*	*S*. Typhi	Incidence of being an *S*. Typhi carrier was higher among schistosomal than non-schistosomal patients
Gendrel D, et al.	1984	Gabon	25	3–18	*S*. *intercalatum*	*S*. Typhi, *S*. Paratyphi A, B, C	*S*. *intercalatum* prolongs *Salmonella* infection
Igwe NN & Agbo EA	2014	Nigeria	500	1–26	*S*. *mansoni*	*S*. Typhi	Strong relationship between *Salmonella* and *S*. *mansoni*; patients with recurrent enteric fever should be screened for asymptomatic schistosomiasis
Mohager MO, et al.	2014	Sudan	288	6–55 (but majority 11–16)	*S*. *mansoni*, *S*. *haematobium*	*Salmonella*	Incidence of *Salmonella* among patients with schistosomiasis higher in children than adults; infection incidence high in those in which schistosomiasis lasted >1 year
Modebe AA, et al.	2014	Nigeria	250	All ages	*S*. *mansoni*	*Salmonella*	*S*. *mansoni* provides place for *Salmonella* multiplication before reentering blood stream; chemotherapy for *Salmonella* must be administered with antischistosomal treatment
Salem AK, et al.	2015	Sudan	203	No ages reported	*S*. *mansoni*, *S*. *haematobium*	*Salmonella*	Direct relationship between schistosomiasis and *Salmonella* infection that requires routine screening in schistosomiasis patients
**Animal studies**								
LoVerde PT, et al.	1980	Lab-based	Hamsters	N/A	*S*. *mansoni*, *S*. *haematobium*, *S*. *japonicum*	*S*. Typhimurium	Pili on *S*. Typhi attaches to schistosome’s digestive opening; nonpiliated may also attached (but reduced rate)
Melhem RF, et al.	1983	Lab-based	Hamsters	N/A	*S*. *mansoni*	*S*. Typhimurium	Pili on *S*. Typhi attaches to schistosome’s digestive opening; *Salmonella* found to only colonize digestive tract due to glycolipids on surface of worm that allows binding
Tuazon CU, et al.	1985	Lab-based	Mice	N/A	*S*. mansoni	*S*. Typhimurium	*Salmonella* bacteremia (regardless of presence of pili) increased mortality rate of mice infected with *S*. *mansoni*; praziquantel administration prior to *S*. Typhi challenge prolonged survival

* Year indicated is year of manuscript publication; no journals indicated the year of data collection, with the exception of the following: Igwe NN & Agbo EA [2012], Salem AK, et al. [2005]

† If subtype mentioned, then identified here; if not, only genus named

‡ Carvalho et al. also included 18 patients who were dually infected with schistosomiasis and Leishmaniasis (but not *Salmonella*)

### Reduced immunological functioning

Both *Salmonella* and schistosomal infection can affect liver and kidney function, which can consequently affect the pharmacokinetics and pharmacodynamics of biological metabolites in the bloodstream [40–42]. Chronic infection of schistosome eggs trapped in the tissues (most commonly in the kidney and liver, though may affect other organs as well) leads to the secretion of proteolytic enzymes that provoke inflammatory and granulomatous reactions. Over time, these reactions inflame the epidermal tissue layer, producing fibrotic deposits that increase the risk of infection by any number of organisms not limited to *Salmonella* [43]. What is unknown, however, is the in vivo nature of the interaction between *Salmonella* and schistosomiasis: Does damage to the liver and kidneys due to schistosomiasis aggravate and prolong *Salmonella* infection, or vice versa?

Previous research examined the extent to which liver infection may affect levels of circulating biochemical compounds in blood serum [34]. In a case-control study of 55 patients (20 of whom were normal controls), El-Hawy et al. found that phagocytosis, a major mechanism within the body’s immune system for removing pathogens and micro-organisms, was affected depending on the patient’s condition. Patients with noncomplicated typhoid who were treated with chloramphenicol exhibited increased phagocytic behavior when compared to normal controls. After the chloramphenicol course, however, phagocytic function did not normalize, but instead was depressed below normal values, suggesting that the drug may affect phagocytosis. Conversely, patients with chronic salmonellosis and *S*. *mansoni* (treated with ampicillin and praziquantel, respectively), and patients with hepatosplenic schistosomiasis and active *S*. *mansoni* (treated with only praziquantel) had depressed levels of phagocytosis—an indication that schistosomiasis may have altered the body’s immune response. Chronic *Salmonella* infection was found to be more difficult to treat in the group with *S*. *mansoni*. Though the mechanism is not fully understood, this may be due to a combination of factors, including multiplication of the *Salmonella* organism hiding within the schistosome and/or altered immune responses that more easily allow the bacteria to multiply inside the reticuloendothelial system cells [34]. More data are needed to better understand how differing antibiotic and antiparasitic treatment regimens affect coinfected patients.

There have also been few studies on coinfection of *Salmonella* and schistosomes that suggest that presence of both conditions further worsens renal function. One Brazilian study analyzed renal involvement of eight patients with hepatosplenic schistosomiasis and prolonged *Salmonella* infection compared to eight patients with schistosomal glomerulopathy (a reduction of the ability of the kidneys to flush out physiologic byproducts from the bloodstream). The authors found that prolonged *Salmonella* infection exacerbates pre-existing schistosomal glomerulopathy due to renal damage caused by the prolonged infection [44]. At least one other study has corroborated this finding: patients with coinfection of both *Salmonella* and schistosomiasis had a greater risk of developing glomerulonephritis, primarily due to *Salmonella* antigens [45].

### Schistosomiasis patients as latent carriers of *Salmonella*

Typhoid fever and iNTS disease have also been found to recur until schistosomiasis is treated, leading some researchers to hypothesize that *Salmonella* is able to persist in the body by attaching to the schistosome. Because of this purported relationship, the *Salmonella* are protected from antibacterial drugs [46–48].

In one of the earliest studies, Young et al. collected *S*. *mansoni* worms from patients with salmonellosis. The samples cultured from the tegument of the worms tested positive for *S*. Paratyphi A. The authors concluded that there exists a symbiotic relationship between *Salmonella* and schistosomes that make dual infection in humans particularly difficult to diagnose and treat [49].

Similar observations have also been made with other species of *Schistosoma*. Expanding on our understanding of this association, LoVerde et al. used some strains of *Salmonella* Typhimurium, a serotype that infects humans as well as some animals, that contained a mutated gene and thus did not express pili (the hair-like appendage found on the surface of bacteria) [50]. Other strains did express pili. Hamsters were infected with various strains of *S*. *mansoni*, *S*. *haematobium*, and *S*. *japonicum* and then sacrificed to remove the adult schistosomes. A subset of the worms’ digestive openings was ligatured to test whether *Salmonella* was associated with the host’s gut and/or with the surface tegument of the worm. The experiment revealed that *S*. Typhimurium was associated with each of the *Schistosoma* tested with the presence of either the pili or the worm’s ligature, though a greater proportion of female worms tested positive for *Salmonella* than did male worms. The presence of both the pili and worm’s ligature increased the likelihood of adhesion between bacterium and parasite when compared to the presence of just one of the appendages. Nevertheless, the study concluded that the pili of the *Salmonella* bacterium, as well as the worm’s digestive tract opening, may play an important role in adhesion of *Salmonella* to the parasite.

This finding was supported by an in vitro experiment that demonstrated that pili expression of the *S*. Typhimurium bacterium is important for attaching to the surface tegument of *S*. *mansoni*. In samples without pili, *Salmonella* was found to only colonize the digestive tract, as opposed to colonizing both the surface tegument (specifically to the mannose-like receptors) and the digestive tract in samples with pili. The mannose-like receptors that bind to mannose, a sugar monomer, were suspected to be glycolipids on the surface of the worms that allow for the binding of the pili of *Salmonella*. This was further confirmed by treating *S*. Typhimurium with antipili antibodies prior to incubation with schistosomes. Because the pili were blocked by antibodies, no bacterial agglutination was observed on the surface of the schistosomes [51].

Tuazon et al. also studied the importance of pili in mice. Mice were infected initially with *S*. *mansoni*; a subset was then either challenged with piliated or nonpiliated *S*. Typhimurium. These mice were compared to another set that was administered praziquantel prior to the *S*. Typhimurium challenge. While mice challenged with nonpiliated bacterium compared to piliated had longer survival times, there was no significant difference in the mortality rates of the mice. However, the study demonstrated that *Salmonella—*regardless of pili presence—increased the mortality rate of mice infected with *S*. *mansoni*. Praziquantel administration prior to *S*. Typhimurium challenge prolonged survival significantly [52]. More studies in humans are needed to better understand the relationship, as well as how the relationship may affect treatment options and detection of schistosomiasis and *Salmonella*.

The synergistic parasite–bacterium relationship in hamsters and mice has been shown in human hosts as well. A number of other studies in Egypt and in Africa have also characterized the relationship between schistosomal infections and urinary typhoid carriers. Because the schistosome eggs cause fibrotic lesions in the urinary tract, typhoid urinary tract infections are persistent and often lead to patients becoming chronic typhoid carriers [35, 53–56]. In one study, patients who were persistent urinary carriers of *Salmonella* did not initially respond to chloramphenicol treatment. Following treatment for bilharzia, however, a second course of the same antibiotic was administered and *Salmonella* was no longer found in urine cultures [55]. Similarly, a study in Gabonese children found that relapse of *Salmonella* infection only occurred in children who were not first treated with niridazole or oltipraz for *S*. *interaclatum* (species that is localized in the colon and rectum). Whether antischistosomal treatment was administered appeared to affect the response to chloramphenicol or ampicillin treatment for *Salmonella* [35]. It will also be important to understand whether protected *Salmonella* bacteria residing inside the schistosomes are shielded from the effects of antibacterial drugs, thereby avoiding the development of drug resistance.

Studies in Nigeria and Sudan have also found a strong, direct relationship between coinfection with *Salmonella* and either *S*. *haematobium* or *S*. *mansoni* [36–39]. One of the studies in Sudan found that the incidence of *Salmonella* was higher among children than adults with schistosomiasis, and that the incidence even increased if an individual (regardless of age) had schistosomiasis for more than a year [37]. As a result of these findings, the authors of these four African studies conclude that patients with recurrent enteric fever living in schistosomiasis endemic regions should also be routinely screened for asymptomatic schistosomiasis. Modebe et al. went further to recommend that chemotherapy for *Salmonella* be administered prior to antischistosomal treatment, as the schistosome creates a place for the *Salmonella* bacterium to multiply before re-entering the bloodstream [38].

## Discussion

The present review suggests an interaction between *Salmonella* and schistosomiasis that will be important to explore further. In spite of the limited number of studies, infection with schistosomes may lead to irreversible damage and residual fibrotic deposits in organs that make one more susceptible to *Salmonella* infections. Further, parasitic infection reduces immunological functioning that makes it more difficult for the body to combat other harmful pathogens, including *Salmonella*. Proper treatment for schistosomes would be advisable prior to further treatment for *Salmonella* both to ensure the effectiveness of medications in eliminating the infection, as well as to prevent further drug resistance. Though, based on the limited evidence, it is not yet clear what the optimal time interval is between infection, treatment, and recovery from either pathogen. For example, a patient is receiving antibiotics for typhoid fever and is infected with schistosomiasis before the course of antibiotics has been completed. Is there a point at which the bacterial load is low enough that the two pathogens do not interact?

These findings have several implications for patients who are coinfected with typhoid and schistosomiasis. Firstly, if a schistosomiasis patient is diagnosed and treated for typhoid using antibiotics, treatment may not be complete—and may inadvertently lead to further resistance in strains of *Salmonella*. Treatment using first-line antibiotics for many *S*. Typhi strains has been decreasing in effectiveness, and many options are no longer available [57–60]. Secondly, incomplete treatment for *Salmonella* due to the schistosome’s shielding effect may give rise to a host of other bacteria control issues and health complications. As the study in Gabonese children discovered, the hidden bacteria residing in the schistosome may be shed, leading to a relapse of *Salmonella*. Consequently, a drug-resistant *Salmonella* strain may proliferate by spreading resistant genes to drug-sensitive bacteria over a prolonged period, resulting in an infection that is much more difficult to treat. Along similar lines, if an individual is coinfected, but unaware of coinfection, and received praziquantel as part of a periodic mass drug administration campaign, it is plausible that killing the schistosomes could lead to a systemic release of hidden *Salmonella*, giving rise to typhoid illness.

As part of its position paper in 2008, WHO recommended that typhoid fever vaccination programs be implemented in the context of other efforts to control the disease [3]. These decisions should be made based on knowledge of the local epidemiology of typhoid—and based on the present review, should also include an understanding of the impact of the interactions between typhoid and other possible pathogens such as schistosomiasis. Despite the availability of licensed typhoid vaccines that offer protection against *S*. Typhi and some Paratyphi, one of which is prequalified by WHO, the number of countries that have incorporated typhoid vaccines into their vaccination programs remains low due to the short efficacy of the vaccines (three years) [61] and the fact that those polysaccharide vaccines cannot be administered to children over two years of age. There is currently no vaccine against the NTS subtypes. Based on the current body of knowledge, it is difficult to conclude whether the effectiveness of a typhoid vaccine would be diminished if adequate treatment and prevention of parasitic infections are not first provided [13]. However, there have been studies that have shown evidence of the reduced protective immune response from vaccines (e.g., tuberculosis, tetanus), in the presence of schistosomiasis [62–64]. There is also no research to our knowledge on whether typhoid vaccination can reduce or stop shedding of *Salmonella* in existing typhoid patients—though typhoid vaccine manufacturers recommend against vaccinating chronic typhoid carriers and individuals with acute infection [65]. Theoretically, vaccinating a typhoid carrier where the *Salmonella* bacterium’s pili is attached to the digestive tract of the worm would most likely not reduce shedding, as it is hidden from the antibodies produced. Consequently, if the vaccine is used in populations with a high burden of typhoid, the continual shedding of the bacterium in spite of vaccination may make the vaccine appear less effective. Understanding the incidence of coinfection between typhoid and schistosomiasis may help in better targeting the typhoid vaccine by prioritizing populations whose immune response would not be compromised by a concurrent schistosomiasis infection.

These key implications must be better understood and assessed. Further coordinated action is needed to improve typhoid surveillance, regulating and promoting appropriate use of antibiotics, and strengthening infection control and prevention plans. While typhoid conjugate vaccines are on the horizon that elicit longer-term protection and can be given to children over two years of age, the most promising vaccine in the pipeline is, at best, two years away from WHO prequalification and widespread use [66]. If we are to have realistic plans for deploying future typhoid vaccines to high-risk populations, we must first understand the true burden of the co-occurrence of *Salmonella* and schistosomiasis and how the interaction between them impacts transmission dynamics.

By using existing surveillance infrastructures in sub-Saharan Africa, we have the opportunity now to study these coinfections in more detail [67]. This is necessary for governments to prioritize interventions—such as vaccine programs and treatment options—that reduce morbidity and mortality. Particularly for children that suffer the highest burdens of both pathogens, breaking the cycle of chronic infection and reinfection with schistosomiasis in order to treat *Salmonella* ensures that children can be healthy enough to go to school and develop physically and mentally in this early stage of life. Schistosomiasis in children has been documented to lead to disabilities including anemia [25], which leads to stunted growth, and poorer short-term memory and mental abilities [68, 69]. Furthermore, because children with schistosomiasis are also likely to live in regions with other endemic helminths and infectious diseases (e.g., malaria), expanding surveillance of other endemic infections in the African region would provide additional opportunities to study the epidemiology of dual- and multipathogen infections. Indeed, some of the studies reviewed found that children and young adults were more likely to be *Salmonella* carriers than older adults [37, 56]. Though beyond the scope of the present review, the interaction between *Salmonella* and schistosomiasis reviewed herein suggests that parasitisms other than schistosomiasis (e.g., lymphatic filariasis, onchocerciasis, loa loa, and other soil-transmitted helminth infections) may have similarly unexplored interactions with *Salmonella*. Because treatment for schistosomiasis and other parasites often overlap, careful consideration should be given to the timing of all antihelmintic treatments.

By studying the combined impact of schistosomiasis and typhoid in Africa, policymakers will be better equipped to understand the nature of the interaction. While this is only one component of comprehensive schistosomiasis and typhoid prevention and control programs, additional data will help ministries of health better target interventions, such as improvements to water, sanitation, and hygiene to cut parasitic and bacterial transmission, praziquantel treatment programs, and targeted vaccinations (for both NTDs). Further, comprehensive strategies that consider the developmental impacts of chronic schistosomiasis will prevent needless prolonged suffering from salmonellosis.
